# Reducing Chronic Visuo-Spatial Neglect Following Right Hemisphere Stroke Through Instrument Playing

**DOI:** 10.3389/fnhum.2014.00413

**Published:** 2014-06-11

**Authors:** Rebeka Bodak, Paresh Malhotra, Nicolò F. Bernardi, Gianna Cocchini, Lauren Stewart

**Affiliations:** ^1^Department of Clinical Medicine, Aarhus University, Aarhus, Denmark; ^2^Division of Brain Sciences, Imperial College London, London, UK; ^3^Department of Psychology, McGill University, Montreal, QC, Canada; ^4^Department of Psychology, Goldsmiths University of London, London, UK

**Keywords:** neglect, stroke, rehabilitation, music therapy, motivation, auditory–motor, spatial attention

## Abstract

Unilateral visuo-spatial neglect is a neuropsychological syndrome commonly resulting from right hemisphere stroke at the temporo-parietal junction of the infero-posterior parietal cortex. Neglect is characterized by reduced awareness of stimuli presented on patients’ contralesional side of space. Inspired by evidence of increased spatial exploration of patients’ left side achieved during keyboard scale-playing, the current study employed a music intervention that involved making sequential goal-directed actions in the neglected part of space, in order to determine whether this would bring about clinically significant improvement in chronic neglect. Two left neglect patients completed an intervention comprising four weekly 30-min music intervention sessions involving playing scales and familiar melodies on chime bars from right to left. Two cancellation tests [Mesulam shape, Behavioral Inattention Test (BIT) star], the neglect subtest from the computerized TAP (Test of Attentional Performance) battery, and the line bisection test were administered three times during a preliminary baseline phase, before and after the four intervention sessions during the intervention phase to investigate short-term effects, and 1 week after the last intervention session to investigate whether any changes in performance would persist. Both patients demonstrated significant short-term and longer-lasting improvements on the Mesulam shape cancellation test. One patient also showed longer-lasting effects on the BIT star cancellation test and scored in the normal range 1 week after the intervention. These findings provide preliminary evidence that active music-making with a horizontally aligned instrument may help neglect patients attend more to their affected side.

## Introduction

Spatial neglect is a frequent consequence of right hemispheric stroke, ranging from 13 to 82%, with a number of studies suggesting that approximately half of these individuals manifest some degree of neglect (Stone et al., 1993; Bowen et al., 1999; Buxbaum et al., 2004; Ringman et al., 2004). It is a heterogeneous neuropsychological syndrome frequently associated with damage to the inferior parietal lobe (Vallar, 1993; Heilman, 2003; Mort et al., 2003), superior temporal gyrus (Karnath et al., 2001), and occasionally with lesions to the white matter tracts (Doricchi et al., 2008).

Demonstrated through a bias toward ipsilesional space, neglect patients present with impaired attention to stimuli located on the contralesional (usually the left) side of the patient’s body and environment. This leads to difficulties engaging in everyday tasks (Luauté et al., 2006; Bowen and Lincoln, 2008), which in turn reduces functional independence. Importantly, the presence of neglect is associated with worse rehabilitation outcome (Jehkonen et al., 2006).

Several rehabilitation techniques have been implemented to reduce neglect. These include training in visual scanning (e.g., Pizzamiglio et al., 2004), prism adaptation (e.g., Rossetti et al., 1998; Humphreys et al., 2006), limb activation (Reinhart et al., 2012), transcutaneous electrical nervous stimulation (TENS) technique (Vallar et al., 1995; Beschin et al., 2012), and virtual reality treatments (Kim et al., 2011; Borghese et al., 2013; see also Rode et al., 2010, for a review). Despite the large body of research investigating treatment techniques for neglect, recent systematic reviews demonstrate a lack of efficacy of existing rehabilitation approaches, with no consensus regarding which technique is most effective (Luauté et al., 2006; Bowen and Lincoln, 2008) and no strong evidence that these approaches lead to improvements in activities of daily living (see for example, review by Barrett et al., 2012).

An intervention based around music-making may hold special promise as a potential new approach. Playing an instrument offers the opportunity to train cognitive and motor skills (Zatorre et al., 2007). For many people, active music-making can be intrinsically rewarding (Altenmüller and Schlaug, 2013), and patients anecdotally report high levels of engagement with rehabilitation exercises that are embedded within a musical context (Bodak, personal communication). The use of music-making as a rehabilitation approach falls under the broad domain of neurologic music therapy (NMT); a neuro-scientifically motivated model of practice, which consists of 20 standardized research-based music therapy techniques (Thaut, 2008). The techniques cover three overarching rehabilitation areas including sensorimotor, speech and language, and cognitive training. As noted in research by Hommel et al. (1990), one of the cognitive training techniques is musical neglect training, which Thaut (2008) defines as a technique that “includes active performance exercises on musical instruments that is structured in time, tempo, and rhythm, and is in appropriate spatial configurations, to focus attention to a neglected or inattended visual field” (p. 196). While evidence on this issue is scarce, one empirical study provides preliminary evidence that making music may ameliorate neglect. Cioffi et al. (2011, 2014) reported that when neglect patients were asked to play consecutive keys on a piano from right to left (into the neglected side), they proceeded further to the left when responses were systematically paired with descending tones, as opposed to random pairing or silence. This improvement may be explained by research findings showing that tasks comprising the active production of a predictable sequence yield better performance (Ishiai et al., 1990, 1997). This suggests that taking advantage of the sequence completion, which is a core component of musical scales and melodies, may facilitate spatial exploration in neglect patients. However, the extent to which this increased spatial exploration might persist and/or translate to improvement on clinical tests of neglect, is unknown.

With this in mind, the aim of the present study was to explore whether a period of active music-making with a horizontally aligned instrument (chime bars) leads to a reduction in attentional bias outside the music session as measured by performance on standard clinical tests for neglect.

## Materials and Methods

### Design

**Figure 1:**
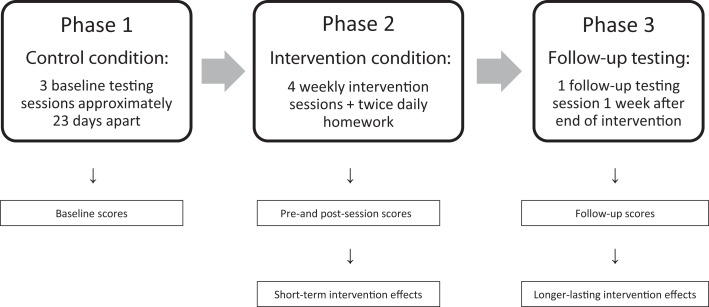
**Outline of experimental design**.

The study followed a within-subject case study design (Figure 1) with participants acting as their own controls. The experiment comprised three phases in the following order: (1) a no-intervention control phase of 6 weeks, during which baseline measures were obtained, followed by (2) an intervention phase of 4 weeks, ending with (3) a single follow-up testing session 1 week after the last intervention session. The dependent variable was performance on tests of visuo-spatial attention, measured within subjects in terms of (a) accuracy on two paper and pencil cancellation tests, (b) accuracy on a computerized target detection task (number of omitted targets in both cases), (c) reaction time on this computer task, and (d) accuracy on the line bisection test (percentage of rightward deviation).

### Materials

Two cancellation tests [Mesulam shape cancellation test, Mesulam, 1985; Star cancellation test as a part of the Behavioral Inattention Test (BIT), Wilson et al., 1987], the neglect subtest from the computerized Test of Attentional Performance (TAP, Zoccolotti et al., 2000) battery, and the line bisection test (Halligan et al., 1990) were administered three times during a 6-week baseline period (Phase 1), before and after each of the four sessions during the intervention phase investigating short-term effects (Phase 2), and at follow-up 1 week after the final intervention session investigating longer-lasting effects (Phase 3).

### Mesulam shape cancellation test

This target detection task is presented to patients on an A4 sheet of paper. It is administered in landscape layout with its center presented to patients at their midline where the researcher sits directly opposite. The test comprises 300 filled and unfilled, familiar (i.e., stars, circles, squares, and triangles), and unfamiliar distractor shapes spatially positioned at random. The target shape is a non-darkened bisected circle with six spines on its outer circumference. Patients are instructed to draw a line through all targets, of which there are a total of 60 with 15 in each quadrant.

### BIT star cancellation test

Like the Mesulam shape cancellation test, this target detection task is also presented to patients on an A4 sheet of paper, in landscape orientation, with the researcher sitting directly opposite. The test comprises 52 darkened large stars, 10 short words, and 13 randomly laid out letters, which are all spread around 56 filled small stars. The targets comprise 54 of the 56 small stars where the two in the center are crossed out by the researcher and excluded from calculations. Patients are instructed to cross out all targets, which are subdivided into 6 sections with 27 on each side.

### Test of attentional performance

In this computerized test, patients are instructed to look at and name letters in the center of the screen throughout the 5-min test. The aim of the task is to detect a total of 44 (11 in each quadrant) stimuli, consisting of successive rapidly changing numbers, by pressing a key. Accuracy and response speed for correctly detected targets is recorded.

### Line bisection

Patients are required to bisect three 180 mm centered horizontal lines on separate landscape A4 sheets of paper.

### Patients

Two outpatients diagnosed with chronic unilateral left-sided spatial neglect following a right hemisphere stroke were recruited to the study. Both patients were right-handed, medically stable, native English-speaking adults who had no known hearing impairment and no prior musical training. Patient 1 had reduced visual acuity in one eye (secondary to previous retinal vein occlusion). Other than this, there was no known prior neurological, cognitive, or psychiatric disease. The study was approved by the National Research Ethics Service and both patients gave full consent.

**Figure 2:**
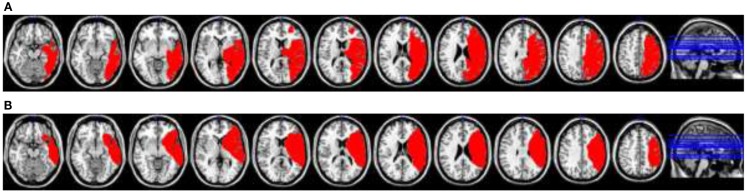
**(A)** Patient 1’s lesion reconstruction (in red) plotted from magnetic resonance imaging acquisitions onto a standard MRI-based template. **(B)** Patient 2’s lesion reconstruction (in red) plotted from magnetic resonance imaging acquisitions onto a standard MRI-based template.

Patient 1 was a 46-year-old male who sustained an ischemic stroke 5 years and 11 months prior to commencement of baseline testing. His stroke resulted in a large right middle cerebral artery territory infarct involving the frontal, parietal, and temporal lobes (Figure 2A). Patient 2 was a 63-year-old male who sustained an ischemic stroke 4 years and 5 months prior to commencement of baseline testing. His stroke also resulted in a large right middle cerebral artery territory infarct involving the frontal, parietal, and temporal lobes (Figure 2B). Patient 2 required a hemicraniectomy involving temporary removal of part of the skull to help reduce brain swelling.

### Procedure

**Figure 3:**
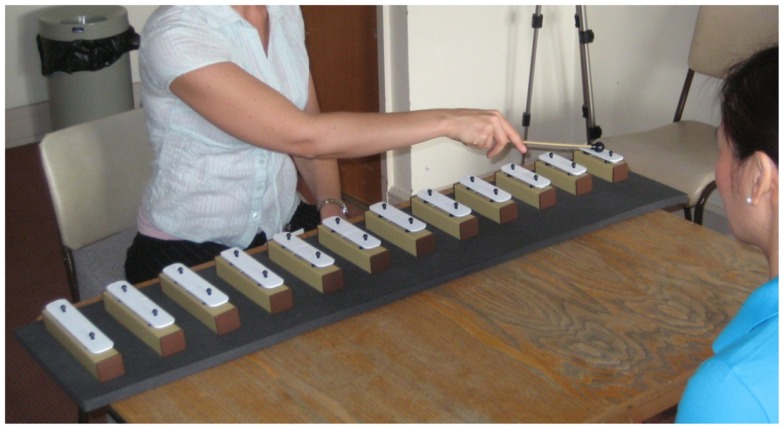
**During the intervention session, the patients observed and repeated the experimenter modeling simple scales and melodies**. A series of three foam frames allowed the spacing between chime bars to be increased as performance improved (see text for more details).

The four weekly 30-min intervention sessions involved playing scales and familiar melodies on 12 chime bars (C_4_–G_5_), which were arranged horizontally, increasing in pitch from right to left (Figure 3). A series of foam frames were used to enable flexibility in the spatial layout using three fixed frame sizes. Chime bars were placed adjacent to each other either (1) one chime bar width apart on the smallest frame (level one), (2) one and a half chime bar widths apart on the middle frame (level two), or (3) two chime bar widths apart on the largest frame (level three). This enabled the intervention sessions to be calibrated for each patient, according to the precise limits of spatial exploration seen. Patients started on level one, and progressed up a level when they played all 12 bars in a row from right to left three consecutive times in one session without errors. When starting at level one, the space between the sixth and the seventh chime bars was at the patient’s midline, with half of the bars reaching to the patient’s right, and the other half reaching to the patient’s left. From this point on, the chime bar at the patient’s far right became the anchor point for successive levels. Namely, it remained in the same place in relation to the patient’s midline throughout the intervention phase, such that the increasing distance between bars stretched out into the left field only, encouraging leftward movement.

Sessions followed an ABA format where part A comprised playing up to one and a half octaves of a C major scale, and part B comprised playing familiar melodies. During the scale-playing, the patient was instructed to “play all the bars, one after the other, right to left, starting here.” At the end of the instruction, the researcher would point to the bar on the patient’s far right to ensure that they started playing at the correct location. The same instruction was repeated three consecutive times both at the start and at the end of each session. Patients were encouraged to play at their own speed.

Both patients reported prior familiarity with the two melodies used in part B – *Frère Jacques* and *Do-Re-Mi*. Both songs start at the first scale degree and slowly move up in chunks of around three (*Frère Jacques* and the first half of *Do-Re-Mi*) to six (the second half of *Do-Re-Mi*) notes at a time.

*Frère Jacques* has a range of six notes (C_4_–A_4_) and each subphrase of the song repeats itself twice, allowing for repetition and encouraging memorization. The benefit of the range was that patients would not be required to cross their midline to complete the song before they had reached level two. Patients progressed to *Do-Re-Mi* after they had played *Frère Jacques* once all the way through from beginning to end with no instruction from the researcher. Self corrected errors were permitted.

*Do-Re-Mi* has a range of an octave; eight white notes (C_4_–C_5_). Therefore, unlike *Frère Jacques*, in order to play the whole song the patient had to cross their midline on all three levels. The subphrases of the second half of the song comprised around six consecutive notes, which encouraged patients to cover a larger spatial array and draw their attention further to their left.

The researcher sat directly opposite the patient throughout the intervention and modeled playing the melodies. This was achieved by initially singing and playing the song that was being worked on from beginning to end, and then breaking it down into small sections for the patient to play back. Each small section was repeated three times before progressing to the next section, to help the patient become familiar with the playing. Each section was then slowly put together, ultimately building it up into a complete piece of music. The patient was invited to sing along throughout.

The scales and familiar melodies that were the focus of each music session were consolidated through structured homework between each session, which was administered via a CD. The patient heard the experimenter verbally explain that they were about to hear the experimenter sing and play a pitch sequence, which they should listen to and repeat. Each exercise corresponded to one CD track. The patient was instructed to complete only the set of exercises (tracks) that were worked on during the intervention session that week, which was clearly written out on a homework sheet. Each patient was provided with a set of chime bars and the foam frame that was appropriate to their respective level at any given week. The patients were asked to work through three sets of the assigned homework exercises twice a day, and to log each completed session.

## Results

### Baseline period

The mean baseline responses from each test are summarized in Table 1. Responses confirmed that both patients scored within the pathological range for neglect across all tests prior to the intervention period with respect to responses made on the left side, as well as in total (left and right side combined). Further, Patient 1’s right side target detection responses on the TAP fell within the pathological range.

**Table 1:** **Baseline descriptive statistics**.

Test				Patient 1		Patient 2	
		Unit	Max. score	Mean	SD	Mean	SD
Mesulam shape	Left	Omissions	30	26	2.65	8^a^	1.00
	Right	Omissions	30	1.33	1.53	0.33	0.58
	Total	Omissions	60	27.33^a^	4.04	8.33^a^	1.53
BIT star	Left	Omissions	27	17.67^a^	2.52	3^a^	2.00
	Right	Omissions	27	1.67	1.53	2	2.65
	Total	Omissions	54	19.33^a^	4.04	5^a^	3.61
TAP	Left	Omissions	22	19^a^	N/A	21^a^	N/A
	Right	Omissions	22	10.67^a^	4.73	1.33	0.58
	Total	Omissions	44	29.67^a^	4.73	22.33^a^	0.58
TAP (mean reaction time)	Left	ms	∞	1276^a^	386.99	N/A	N/A
	Right	ms	∞	869	135.35	903.67	113.32
	Total	ms	∞	1073^a^	174.79	946.70^a^	66.32
Line bisection^b^		mm		10.22^a^	6.43	18.56^a^	9.14

*^a^ Within pathological range as reported in previous studies: Mesulam shape (Mesulam, 1985; Machner et al., 2012), BIT Star (Wilson et al., 1987), TAP (Zimmermann and Fimm, 2007), line bisection (Halligan et al., 1990; Mort et al., 2003)*.

*^b^ Average rightward deviation from true center from nine separate 180 mm lines (three each testing session)*.

*N/A, not available; unable to compute as either no variation between baseline scores (left TAP omissions) or unavailable mean reaction times resulting from either zero or only one detected stimuli on the left side*.

### Short-term and longer-lasting treatment effects

To determine whether significant short-term changes in performance had occurred as a function of the intervention sessions, one sample *t*-tests were conducted using change scores (calculated by subtracting the pre-intervention scores from post-intervention scores) for each patient’s left side and total (combined left and right) responses on each of the four tests. To test for longer-lasting treatment effects, *z*-scores were calculated, comparing scores on each test at follow-up for each patient against the corresponding mean baseline scores.

A summary of significant short-term (average pre and average post) and longer-lasting (mean baseline and follow-up) improvement can be seen for each patient in Table 2.

**Table 2:** **Summary of results**.

Test		Unit	Patient 1				Patient 2			
			Average	Average	Mean	Follow-up	Average	Average	Mean	Follow-up
			pre	post	baseline		pre	post	baseline	
Mesulam shape	Left	Omissions	**24**	**13**	**26**	**11**	**5**	**2**	**8**	**1**
	Total	Omissions	**27**	**13**	**27**	**13**	**6**	**2**	**8**	**1**
BIT star	Left	Omissions	8	6	**18**	**8**	5	3	3	1
	Total	Omissions	10	7	**19**	**10**	6	3	5	1
TAP	Left	Omissions	21	20	19	19	21	21	21	20
	Total	Omissions	29	27	30	23	22	22	22	24
Line bisection^a^		mm	11	14	10	17	20	10	19	16

*Bold typeface indicates a significant improvement*.

*^a^Average rightward deviation from the true center from three separate 180 mm lines during each testing session*.

### Mesulam shape cancellation test: Patient 1

**Figure 4:**
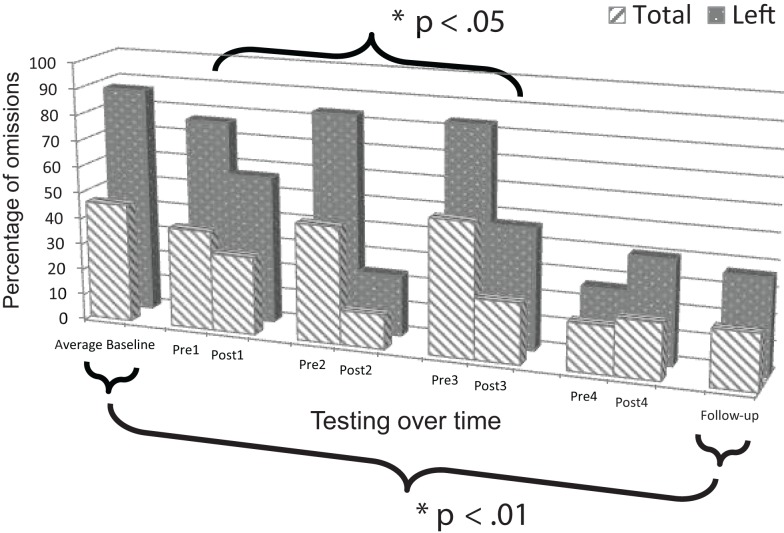
**Plot of Patient 1’s average baseline, intervention period, and follow-up responses on the *Mesulam shape cancellation test***.

Change scores for post versus pre-intervention sessions revealed a non-significant trend, both for responses on the left side, *t*(3) = 1.675, *p* = 0.096, and in total, *t*(3) = 1.954, *p* = 0.073 (Figure 4). An apparent lack of change post versus pre-intervention for session four appeared to be attributable to a ceiling effect resulting from a lasting improvement between the end of session three and the start of session four. When session four was omitted from the analysis, performance post versus pre-intervention for the remaining three sessions was significantly improved, both for responses on the left side, *t*(2) = 3.352, *p* = 0.040, and in total, *t*(2) = 3.126, *p* = 0.045.

Comparison of mean baseline performance with performance at follow-up showed significant improvement, both for responses on the left side, *z* = 5.76, *p* < 0.001, and in total, *z* = 3.55, *p* < 0.001 (Figure 4).

### Mesulam shape cancellation test: Patient 2

**Figure 5:**
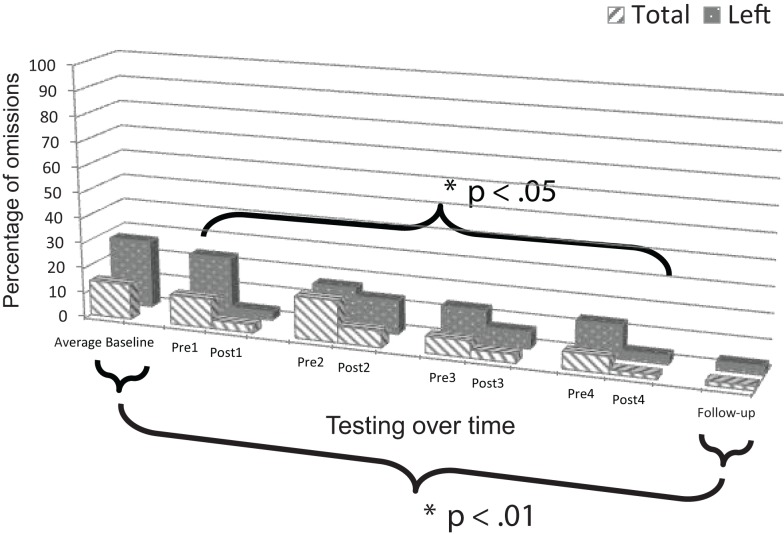
**Plot of Patient 2’s average baseline, intervention period, and follow-up responses on the *Mesulam shape cancellation test***.

Change scores for post versus pre-intervention sessions revealed significant improvement, both for responses on the left side, *t*(3) = 2.777, *p* = 0.035, and in total, *t*(3) = 4.382, *p* = 0.011 (Figure 5). Additionally, performance was in the normal range as evidenced by omitting a total of two or fewer targets after sessions one (Post1), three (Post3), and four (Post4).

Comparison of mean baseline performance with performance at follow-up showed significant improvement, both for responses on the left side, *z* = 7.00, *p* < 0.001, and in total, *z* = 4.79, *p* < 0.001 (Figure 5). Moreover, at the follow-up session, performance was in the normal range on the test as evidenced by omitting a total of only one target.

### BIT star cancellation test: Patient 1

**Figure 6:**
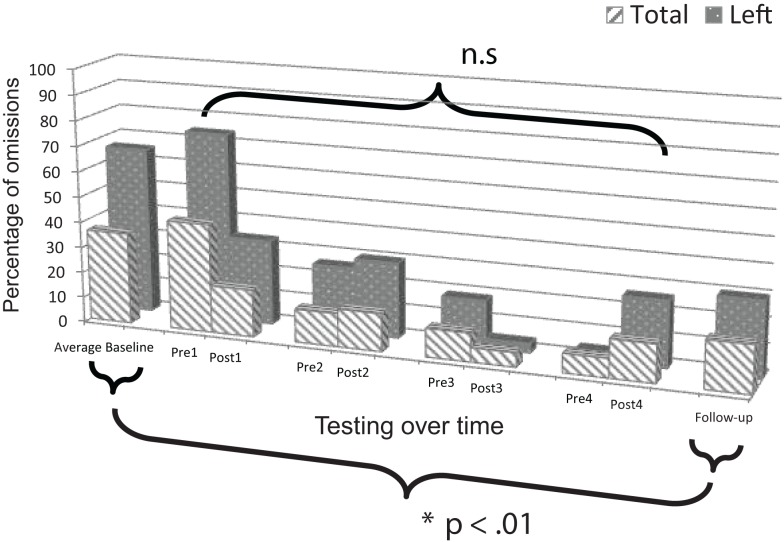
**Plot of Patient 1’s average baseline, intervention period, and follow-up responses on the *BIT star cancellation test***.

Change scores for post versus pre-intervention sessions did not reveal significant improvement, neither for responses on the left side, *t*(3) = 0.551, *p* = 0.310, nor in total, *t*(3) = 0.742, *p* = 0.256 (Figure 6).

Comparison of mean baseline performance with performance at follow-up showed significant improvement, both for responses on the left side, *z* = 3.84, *p* < 0.001, and in total, *z* = 2.31, *p* = 0.021 (Figure 6).

### BIT star cancellation test: Patient 2

**Figure 7:**
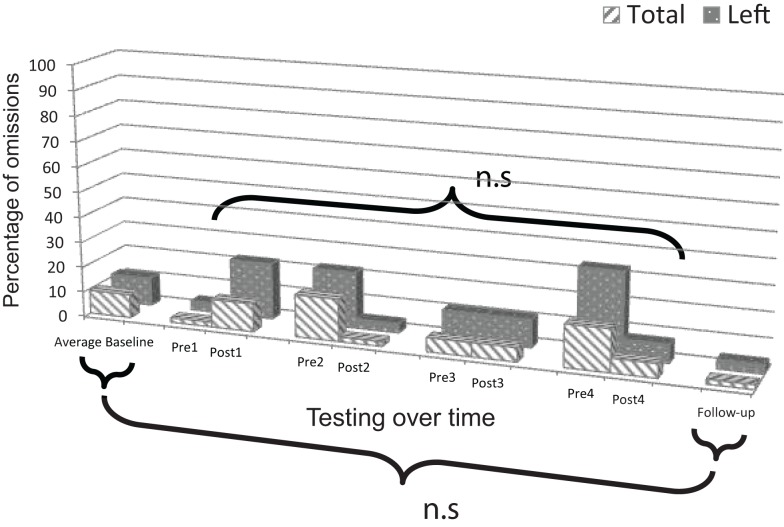
**Plot of Patient 2’s average baseline, intervention period, and follow-up responses on the *BIT star cancellation test***.

Change scores for post versus pre-intervention sessions did not reveal significant improvement, neither for responses on the left side, *t*(3) = 0.651, *p* = 0.281, nor in total, *t*(3) = 0.762, *p* = 0.762 (Figure 7).

Comparison of mean baseline performance with performance at follow-up did not show significant improvement, neither for responses on the left side, *z* = 1.00, *p* = 0.317, nor in total, *z* = 1.12, *p* = 0.263 (Figure 7). Performance was in the normal range on the test as evidenced by omitting a total of only one target three times during the study including before session one (Pre1), after session two (Post2), and at follow-up.

### Test of attentional performance (TAP)

Although response time data were collected, mean response times could not be computed owing to a paucity of correctly detected targets in both patients.

### TAP (omissions): Patient 1

Change scores for post versus pre-intervention sessions did not reveal significant improvement, neither for responses on the left side, *t*(3) = 0.577 *p* = 0.302, nor in total, *t*(3) = 0.739, *p* = 0.257.

Analysis of longer-lasting effects could not be computed for responses on the left side because of insufficient variation in performance during the baseline period (omissions scores were identical for all three baseline testing sessions). Comparison of mean baseline performance with performance at follow-up did not show significant improvement on total test responses, *z* = 1.41, *p* = 0.159.

### TAP (omissions): Patient 2

Change scores for post versus pre-intervention sessions did not reveal significant improvement, neither for responses on the left side, *t*(3) = −1.000, *p* = 0.196, nor in total, *t*(3) = −0.293, *p* = 0.395.

As for Patient 1, analysis of longer-lasting effects could not be computed for responses on the left side because of insufficient variation in performance during the baseline period (omissions scores were identical for all three baseline testing sessions). Comparison of mean baseline performance with performance at follow-up showed significant decline in performance on total test responses, *z* = −2.88, *p* = 0.004, attributable to a change in performance on the right side.

### Line bisection test: Patient 1

Change scores for post versus pre-intervention sessions did not reveal significant improvement on test responses, *t*(3) = −2.189, *p* = 0.058.

Comparison of mean baseline performance with performance at follow-up did not show significant improvement on test responses, *z* = −1.11, *p* = 0.267.

### Line bisection test: Patient 2

Change scores for post versus pre-intervention sessions did not reveal significant improvement on test responses, *t*(3) = 1.242, *p* = 0.151.

Comparison of mean baseline performance with performance at follow-up did not show significant improvement on test responses, *z* = 0.028, *p* = 0.780.

## Discussion

Operating within the broad framework of NMT (Thaut, 2008), the aim of the present study was to explore whether musical training on a horizontally aligned instrument (chime bars) would increase spatial awareness in patients with chronic unilateral neglect. It was hypothesized that patients would perform better on neglect tests after, compared to before, the music intervention sessions (demonstrating short-term treatment effects), and that patients would perform better on neglect tests at follow-up one week after the intervention compared to the baseline period (demonstrating a longer-lasting treatment effect).

As predicted, short-term treatment effects were found for both participants on the Mesulam shape cancellation test. For Patient 1, improvement was observed after sessions one, two, and three. At the beginning of sessions two and three, however, performance returned to a similar level seen at baseline. Interestingly, this fluctuation seemed to stabilize after session three, which may be explained by a consolidation of repeated effects of the intervention and weekly homework. While no significant short-term improvements were seen on the BIT star cancellation test for either patient, for Patient 1 at least, this may be explained due to a large and sustained treatment effect following session one, leaving little room for further short-term improvements.

Longer-lasting treatment effects were found for both participants on the Mesulam shape cancellation test. Patient 1 showed this longer-lasting treatment effect on the BIT star cancellation test, despite not showing significant short-term improvements (supporting the above suggestion that an early and sustained improvement occurred in session one). Furthermore, Patient 2 performed in the clinically normal range on both the Mesulam shape and BIT star cancellation tests at follow-up.

An important possibility to consider is whether or not the improvements seen during the intervention period may be at least in part owing to the repeated testing and hence increased familiarity with the clinical tests. We consider this unlikely for two reasons: first, neither patient showed systematic improvement during the baseline period on either of the cancellation tasks, which would be expected according to a “practice effect” explanation. Second, a recent study examining test–retest reliability of two cancellation tasks, including the Mesulam cancellation task, in 15 chronic neglect patients over five consecutive days, demonstrated stable performance that did not show a significant practice effect (Machner et al., 2012).

While significant improvements were observed on the Mesulam shape cancellation test, and to some extent on the BIT star cancellation test, similar improvements were not found on the TAP or the line bisection. One reason for the improved performance on the paper and pencil cancellation tasks may be that these tasks most closely mirror the nature of the intervention, whereby patients were required to constantly orient their attention and make movements toward their left side in order to find the next chime bar to play. Moreover, the cancellation tasks, like the music training, involved sequential processing of information, rather than repeated responses in the same location as was required for the line bisection and TAP. Thus, there might be a more effective transfer of “trained” behavior contributing to this finding.

Clearly, many aspects of the musical intervention used here may have contributed to the improvements seen in both patients and further studies may attempt to isolate some of the potential underlying mechanisms of the intervention. Music-making of the kind involved here is by nature visuomotor and goal-directed, both of which have been highlighted as important factors for rehabilitation of spatial attention (Harvey et al., 2003; Harvey and Rossit, 2012). In addition, the sequential aspect of the intervention, whereby patients played back simple and short scales and melodies with a predetermined sequence and clear end-point is likely to have played a role, echoing Ishiai and colleagues’ findings, which showed that patients performed better on tasks of visuo-spatial attention when such tasks required them to sequentially number targets (1990) and complete sequences (1997). Further, the spatially systematic nature of the pitch-based feedback may have also played an important role: Cioffi et al. (2011, 2014), showed that patients with neglect proceeded further to the left while playing a keyboard when responses were systematically paired with tones, as opposed to random pairing of key/pitch or silence. Determining the relative contributions of the pitch feedback versus the role of making repeated physical movements in the neglected part of space could be tested by comparing (1) the current combined auditory–motor intervention against (2) a motor only intervention with silent chime bars or woodblocks that produce sound with no changing pitch against (3) an auditory only intervention comprising vertical or stationary playing at midline, with pitch feedback.

As with any intervention approach where lasting effects are desired, it will be important to determine the dose–response relationship: how many sessions are optimal to achieve lasting treatment effects, how long do such effects last, and do they translate into improvement in activities of daily living, as measured by more ecological tasks such as the Catherine Bergego Scale (Azouvi, 2003). While both patients in the study had chronic neglect, further work will be required to ascertain whether such an intervention can improve neglect in the acute stage. Similarly, while neglect is typically a heterogeneous syndrome (Hillis, 2006), our findings are most relevant to patients with peripersonal neglect and it remains an empirical question as to whether the benefits would extend, e.g., to presentations of auditory or personal neglect.

The current study has demonstrated that active music-making holds promise as an effective intervention for neglect patients. Further research in this as yet untapped area has key implications for the improvement of existing clinical interventions and development of future treatment protocols. A deeper understanding of the underlying mechanisms has the capacity to contribute to the expanding scientific knowledge base of music interventions relevant to improving quality of life in this clinical group.

## Conflict of Interest Statement

The authors declare that the research was conducted in the absence of any commercial or financial relationships that could be construed as a potential conflict of interest.
